# Levels of pathogen virulence and host resistance both shape the antibody response to an emerging bacterial disease

**DOI:** 10.1038/s41598-021-87464-9

**Published:** 2021-04-15

**Authors:** Daisy E. Gates, Molly Staley, Luc Tardy, Mathieu Giraudeau, Geoffrey E. Hill, Kevin J. McGraw, Camille Bonneaud

**Affiliations:** 1 Centre for Ecology and Conservation, University of Exeter, Penryn, Cornwall, TR10 9FE UK; 2 Department Biological Science, Auburn University, Auburn, Alabama, 36849-5414 USA; 3 School of Life Sciences, Arizona State University, Tempe, AZ 85287-4501 USA; 4 Present Address: Biology Department, Loyola University Chicago, Chicago, IL 60660-1537 USA; 5 Present Address: Centre for Ecological and Evolutionary Research On Cancer, UMR CNRS/IRD/UM 5290 MIVEGEC, 34394 Montpellier, France

**Keywords:** Behavioural ecology, Ecological epidemiology, Ecophysiology, Ecology

## Abstract

Quantifying variation in the ability to fight infection among free-living hosts is challenging and often constrained to one or a few measures of immune activity. While such measures are typically taken to reflect host resistance, they can also be shaped by pathogen effects, for example, if more virulent strains trigger more robust immune responses. Here, we test the extent to which pathogen-specific antibody levels, a commonly used measure of immunocompetence, reflect variation in host resistance versus pathogen virulence, and whether these antibodies effectively clear infection. House finches (*Haemorhous mexicanus*) from resistant and susceptible populations were inoculated with > 50 isolates of their novel *Mycoplasma gallisepticum* pathogen collected over a 20-year period during which virulence increased. Serum antibody levels were higher in finches from resistant populations and increased with year of pathogen sampling. Higher antibody levels, however, did not subsequently give rise to greater reductions in pathogen load. Our results show that antibody responses can be shaped by levels of host resistance and pathogen virulence, and do not necessarily signal immune clearance ability. While the generality of this novel finding remains unclear, particularly outside of mycoplasmas, it cautions against using antibody levels as implicit proxies for immunocompetence and/or host resistance.

## Introduction

Within any population of host, individuals typically vary in their ability to fight infection. Although understanding this variation is key to predicting host–pathogen interactions, from epidemiological dynamics to their co-evolutionary trajectories, quantifying it in free-living host populations presents a serious challenge^1–4^. Often, it is only possible to take one or a few measurements of immune molecules or cells that are then used as proxies of an individual’s ability to clear infection^2^. There are reasons to question, however, whether such proxies are always good predictors of resistance. For instance, variation in the immune responses measured may, instead, reflect the level of virulence of the pathogen strain, with more virulent strains eliciting more robust responses^5–8^. Furthermore, across infected individuals, the immune responses measured may not signal clearance ability if their primary role is to prevent infection establishment, or if different pathogen strains vary in their ability to evade immunity^9^. While differences in the immune response to a given pathogen clone are likely to reflect among-host variation in resistance, any natural variation in the pathogen, including in its virulence, may complicate the relationship between immune activity and resistance. Determining the effectiveness and adaptive benefits of immune activity will, as a result, require functional tests that are conducted across a range of different pathogen strains.

Circulating levels of antibodies (immunoglobulins) are often used to estimate immunocompetence in natural populations because they can be relatively easily measured^10^. Antibodies are produced by B lymphocytes in response to specific antigens and can control invading pathogens through various mechanisms, including neutralization, prevention of adherence, enhancement of phagocytosis through opsonisation or activation of the complement cascade^11^. In natural populations of Soay sheep (*Ovis aries*), for example, total nematode-specific antibody levels in blood plasma were found to negatively correlate with parasite egg counts in the faeces of lambs over a 25-year period, suggesting that antibody levels are a useful metric of resistance in this system^12,13^. However, antibody levels against the fungal disease *Pseudogymnoascus destructans* were found to be lower in two European *Myotis* bat species that have coevolved with the pathogen than in newly exposed North American little brown bats (*Myotis lucifugus*), suggesting that, in this case, the antibody response is not consistently an accurate predictor of survival to infection^14^. Together these studies highlight the complex relationship between antibody responses and their protective benefits to hosts.

We can use the recently established host–pathogen system consisting of the house finch (*Haemorhous mexicanus*) from North America and its conjunctivitis-causing bacterium, *Mycoplasma gallisepticum*, to test the role of host resistance versus pathogen virulence in shaping among-host variation in immune activity. *M. gallisepticum* emerged in house finches in 1994 following a single host shift from poultry^15–17^, causing a devastating epidemic that spread rapidly throughout the eastern United States (U.S.)^18, 19^. In disease-exposed populations, the intensity of pathogen-induced selection led to the spread of resistance from standing genetic variation within 12 years of epidemic outbreak^20, 21^, although some western populations have remained unexposed to present day^22^. The evolution of host resistance, in turn, favoured the evolution of increasing pathogen virulence^21^, with late-epidemic isolates causing greater putative host mortality and more severe signs of conjunctivitis than earlier ones^23^. As a result, this system presents the necessary variation in host resistance and pathogen virulence required for testing the contribution of each in explaining variation in the immune response to infection.

Here, we conducted a test on the systemic antibody (immunoglobulin Y–IgY) response of house finches to *M. gallisepticum*, which is commonly used to determine past or current infections^16, 24^. IgYs are the most abundant circulating antibody isotype in birds, reptiles and amphibians, and the functional equivalent of mammalian IgG^25^. This antibody class immobilises pathogens through binding via agglutination or coating pathogen surfaces (referred to as opsonisation), and can also activate the complement cascade^26^. We measured serum levels of *M. gallisepticum*-specific IgYs in samples obtained from an infection experiment in which house finches from *M. gallisepticum*-exposed populations that have evolved resistance, as well as from unexposed populations that are susceptible, were inoculated with *M. gallisepticum* isolates of varying virulence and collected over a 20-year period from outbreak^21^. A previous study showed that, following inoculation, finches from resistant and susceptible populations were just as likely to become infected and displayed similar pathogen loads 8 days post-inoculation, but that finches from resistant populations subsequently cleared the infection^27^.

In our experiment, > 50 pathogen isolates sampled in 9 separate years (1–9 isolates collected per year over a 20-year period) were each inoculated into two house finches, one from resistant and one from susceptible populations. Doing so allowed us to test the extent to which host resistance and pathogen virulence shape the antibody response using a regression-based approach. We favoured a regression-based approach (i.e. maximising the number of isolates) over a replicate-based approach (i.e. maximising the number of replicates per isolate), because we wished to encapsulate sufficient natural variation in both host resistance and pathogen virulence to quantify statistically the relative impacts of each in shaping antibody levels. This aim is at odds with the alternative replicate-based approach for two reasons. First, while the replicate-based approach is preferred for clarifying differences among a limited set of isolates (e.g. A vs. B vs. C), it is not appropriate for quantifying the relative explanatory power of two or more predictors. Second, since in naturally evolving systems one can only replicate the pathogen isolate, there is a danger of spuriously increasing the relative importance of pathogen traits relative to host traits, as replicates will necessarily reduce natural variation and measurement error. Nevertheless, we maximised the power of our regression-based approach using a paired-experimental design in which each isolate was inoculated into two house finches of the same age (i.e. ~ 6 months old) and that had never been exposed to the pathogen previously, but with one originating from resistant and the other from susceptible populations (see “Methods” section).

We then tested the following hypotheses. (1) We tested whether infection intensity (i.e. pathogen load) was associated with serum levels of *M. gallisepticum*-specific antibodies. Because pathogen load is an outcome of the host–pathogen interaction, we then tested whether any association between pathogen load and antibody levels was driven by differences in host resistance and/or pathogen virulence. Antibody levels would reflect resistance if finches from resistant populations displayed higher levels of specific IgYs than finches from susceptible populations. By contrast, antibody levels would reflect variation in pathogen virulence if late-epidemic isolates (i.e. those that are more virulent) elicited higher antibody responses than early-epidemic ones. (2) We then tested the extent to which antibody responses were associated with a change in pathogen load over the course of the infection. Here antibody levels would be indicative of immune clearance ability if individuals displaying higher antibody responses subsequently exhibited the greatest decline in pathogen load.

## Results

### Antibody levels and associating factors

**Figure 1:**
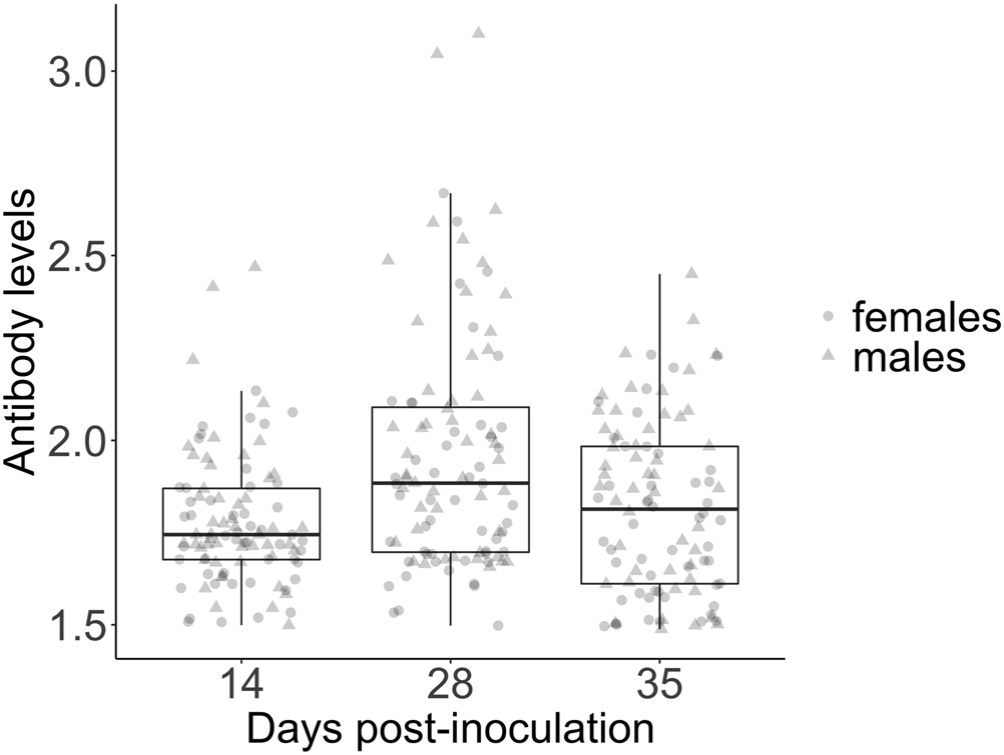
Variation in antibody levels in both sexes over the course of the infection. We measured antibody levels (in ELISA Units (EU)/ml) at 14, 28 and 35 days post-inoculation (dpi). Boxplots show the median and inter-quartile range, whiskers show maximum values and points represent raw data (dots = females, triangles = males).

Mean antibody level over the course of the experiment was 1.85 ± 0.26 ELISA Units (EU)/ml (range 1.49–3.10). Antibody levels were highest at 28 dpi and more comparable on 14 and 35 dpi (linear mixed model; *χ*^2^ = 43.2, *p* < 0.0001; estimate (14 vs. 28 dpi) ± SE = 0.09 ± 0.01; estimate (14 vs. 35 dpi) ± SE = 0.02 ± 0.01; Figs. 1 and S1). Despite changes in antibody levels between 14 and 35 dpi, antibody levels remained weakly but significantly repeatable within individuals (R = 0.38 ± 0.06; likelihood ratio test = 201.7, *p* < 0.0001; birds from susceptible populations only: R = 0.38 ± 0.09; birds from resistant populations only: R = 0.38 ± 0.09). Although we found no effect of body mass on antibody levels on any of the day measured, sex significantly associated with antibody levels at 28 and  35 dpi, with males displaying significantly higher antibody levels on those days than females (Table 1). As a consequence, all statistical results are provided with sex included as a cofactor.

**Table 1:** Effects of sex and body mass on antibody levels at 14, 28 and 35 dpi.

Response term	Explanatory term	Estimate ± SE	t statistics	*p* value
Antibodies at 14 dpi	Sex	0.05 ± 0.04	t_2,96_ = 1.3	0.19
	Initial body mass	0.02 ± 0.015	t_2,96_ = 1.4	0.16
Antibodies at 28 dpi	Sex	0.06 ± 0.02	t_2,97_ = 2.7	0.008*
	Initial body mass	− 0.004 ± 0.009	t_2,97_ = − 0.5	0.64
Antibodies at 35 dpi	Sex	0.04 ± 0.02	t_2,97_ = 2.5	0.014*
	Initial body mass	− 0.009 ± 0.007	t_2,97_ = − 1.2	0.23

We ran linear models with antibody levels at 14 dpi or sqrt(antibody levels at 28 or 35 dpi) as the response term and with sex and body mass at the start of the experiment as explanatory terms.

**Figure 2:**
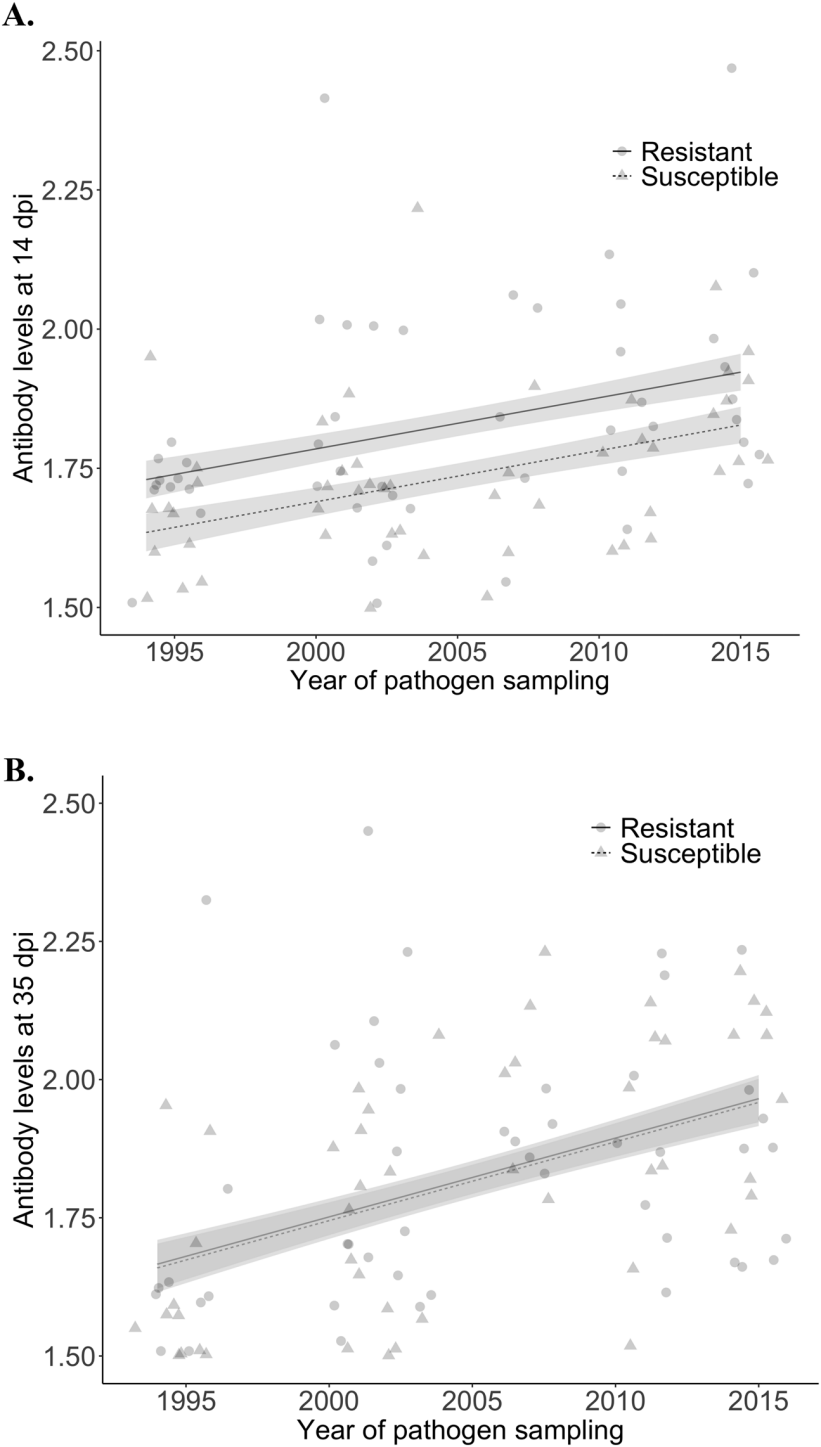
Association between antibody levels (in EU/ml), year of pathogen sampling (1994–2017) and host population (resistant and susceptible) at (**A**) 14 dpi, and (**B**) 35 dpi (statistics provided in Table 2). Points represent raw values; lines are predicted from the model (solid = resistant host populations; dashed = susceptible host populations), with standard errors represented by ribbons. Note that points were jittered for clarity.

We tested whether infection intensity (i.e. pathogen load) predicted subsequent antibody levels, and whether any effect of pathogen load was driven by differences in host resistance and/or pathogen virulence. We found that bacterial load at 8 dpi significantly predicted antibody levels at 14 dpi (linear mixed model; estimate ± SE = 0.0007 ± 0.0002, *χ*^2^ = 7.7, *p* = 0.005). To investigate the contribution of variation in host resistance and/or pathogen load in driving the association between bacterial load and antibody levels, we fitted whether the hosts were from susceptible or resistant populations^27^ (hereafter: host resistance status) and year of pathogen sampling ^23^ as our explanatory variables, since all other metrics of infection (e.g. clinical symptoms severity) will reflect the outcome of the interaction between host resistance and pathogen virulence. The association between bacterial load at 8 dpi and antibody levels at 14 dpi was explained by a significant effect of both host resistance status and year of pathogen sampling (Table 2). Indeed, antibody levels at 14 dpi were significantly higher in birds from resistant relative to susceptible populations (resistant = 1.8 ± 0.2 EU/ml; susceptible = 1.7 ± 1.4 EU/ml), and they increased significantly as the pathogen was sampled progressively later in the epidemic and hence became more virulent (Fig. 2; Table 2).

**Table 2:** Effects of host resistance and pathogen virulence on antibody levels.

Response term	Explanatory term	Estimate ± SE	*χ*^2^	*p* value
Antibody levels at 14 dpi	Bacterial load at 8 dpi	0.0004 ± 0.0003	2.1	015
	Host resistance status	− 0.09 ± 0.03	8.4	0.004*
	Pathogen year	0.007 ± 0.003	8.2	0.004*
	Sex	0.03 ± 0.03	1.0	0.3
Antibody levels at 35 dpi	Bacterial load at 28 dpi	0.0007 ± 0.0002	20.3	< 0.0001*
	Host resistance status	− 0.01 ± 0.01	1.0	0.33
	Pathogen year	0.003 ± 0.001	8.3	0.004*
	Sex	0.04 ± 0.01	8.3	0.004*

We ran linear mixed models with either antibody levels at 14 dpi or sqrt(antibody levels at 28 or 35 dpi) as the response term, and with pathogen load at 8, 21 or 28 dpi, host resistance status (resistant vs. susceptible populations) and the year of pathogen sampling (1994–2015) as explanatory terms; sex was included as a cofactor and pathogen isolate identity as a random term.

These results were largely mirrored at subsequent time points. While bacterial load at 21 dpi did not predict antibody levels on day 28 dpi (linear mixed model; estimate ± SE = 0.0002 ± 0.0002, *χ*^2^ = 1.6, *p* = 0.20), bacterial load at 28 dpi significantly and positively predicted antibody levels at 35 dpi (linear mixed model; estimate ± SE = 0.0008 ± 0.0001, *χ*^2^ = 26.2, *p* < 0.0001). In addition, antibody levels at 35 dpi were significantly associated with the year of pathogen sampling but not host resistance status (Table 2), with a similar effect of year of pathogen sampling in resistant and susceptible host populations (linear mixed model; year of pathogen sampling × host resistance status interaction effect: estimate ± SE = 0.002 ± 0.002, *χ*^2^ = 1.4, *p* = 0.23). Taken together, our results indicate that bacterial load shaped subsequent antibody levels, and that the host resistance status and the year of pathogen sampling both drove this association. Indeed, although individuals from both resistant and susceptible populations were able to mount an antibody response, the response of birds from resistant populations was greater than that of birds from susceptible populations. Further, antibody levels increased as the pathogen isolate was sampled later in the epidemic and was, hence, more virulent.

### Antibodies levels and immune clearance ability

We tested whether antibody responses indicated immune clearance ability and gave rise to a subsequent decline in pathogen load. A main effect of antibody levels at 14 dpi on the change in bacterial load between 14 and 21 dpi would not suggest causation because of the correlations that exist between antibody levels at 14 dpi and bacterial load at 8 dpi (see above). By contrast, causation would be suggested if individuals exhibiting the same pathogen load, but differing antibody levels at 14 dpi, exhibited contrasting changes in bacterial load between 14 and 21 dpi. For example, antibody levels would suggest immune clearance ability if individuals with high bacterial loads and high antibody levels at 14 dpi showed a reduction in bacterial load between 14 and 21 dpi that was greater than those with high bacterial loads and low antibody levels at 14 dpi. However, we found no evidence that the antibody response at 14 dpi was indicative of the change in bacterial load between 14 and 21 dpi (Table 3). Similarly, we found no evidence that the antibody response at 14 dpi was indicative of the change in bacterial load between 14 and 28 dpi (Table 3). Our measures of antibody levels therefore do not appear reflective of the ability of house finches to clear *M. gallisepticum* infection.

**Table 3:** Effects of antibody levels on changes in pathogen load.

Response term	Explanatory term	Estimate ± SE	*χ*^2^	*p* value
Change in bacterial load between 14 and 21 dpi	Antibody levels at 14 dpi	− 13.1 ± 31.1	1.3	0.26
	Bacterial load at 14 dpi	0.03 ± 0.61	89.4	< 0.0001*
	Antibody levels at 14 dpi × bacterial load at 14 dpi	− 0.35 ± 0.32	1.3	0.26
	Sex	− 10.6 ± 9.5	1.1	0.29
Change in bacterial load between 14 and 28 dpi	Antibody levels at 14 dpi	1.3 ± 29.9	0.03	0.86
	Bacterial load at 14 dpi	− 0.58 ± 0.58	12.8	< 0.0001*
	Antibody levels at 14 dpi × bacterial load at 14 dpi	− 0.11 ± 0.3	0.1	0.71
	Sex	− 6.4 ± 10.2	0.34	0.56

We ran linear mixed models with the difference in pathogen load between 21 and 14 dpi or between 28 and 14 dpi as the response term, and with antibody levels at 14 dpi, pathogen load at 14 dpi and their interaction as explanatory terms; sex was included as a cofactor and pathogen isolate identity as the random term.

## Discussion

By experimentally inoculating house finches from coevolved (resistant) and unexposed (susceptible) populations with pathogen isolates sampled over 20-year of the epidemic during which virulence is known to have increased^23^, we were able to test the roles of host resistance and pathogen virulence in shaping the antibody response. We found that all finches mounted an antibody response and that finches from resistant populations mounted a significantly higher antibody response than those from susceptible populations. In addition, antibody levels increased positively with year of pathogen sampling. In other words, although antibody levels should reflect levels of resistance when all birds are exposed to the same isolate, the association between antibody levels and resistance will be obscured when multiple pathogen isolates of differencing virulence are involved. Finally, we found that antibody levels were, however, not indicative of immune clearance ability. Indeed, there was no evidence to suggest that antibody levels on our measurement days shaped pathogen load because, for a given level of pathogen load, individuals with higher antibody levels were not subsequently better able to clear the infection. Taken together, our results suggest that antibody production is reflective of both the levels of host resistance and pathogen virulence, and cannot, in our case, be used as a proxy for immune clearance ability.

Our results are unlikely to be confounded by differences in the birds’ prior exposure to the pathogen over their short life before capture, or by genetic differences between the isolates of *M. gallisepticum* used. First, regarding the potential issue of differences in prior exposure, we ensured that (a) none of the birds used in this study was infected with *M. gallisepticum* at the time of capture; and that (b) none had *M. gallisepticum*-specific antibodies before experimental onset. Together, these indicate that none of the birds had been previously exposed and that the antibody response to experimental inoculation was a primary, not a secondary, response to infection. Second, differences in the *M. gallisepticum*-specific IgY levels detected using our method are unlikely to be shaped by genetic differences between isolates for three reasons. (1) Coating the plate microwells with inactivated whole *M. gallisepticum* of poultry origin provides numerous antigens with which house finch antibodies can interact, thereby reducing the likelihood that variation in antibody levels will reflect differences in the ability to cross-react and bind the coated antigens. (2) Using the same methodology, we previously showed that house finches inoculated with chicken and house finch *M. gallisepticum* both mounted detectable antibody responses, but the response to the house finch isolate was greater—a pattern which is opposite to the one expected if the assay had been preferentially biased in favour of antibodies directed against chicken *M. gallisepticum*^28^. (3) If antibody levels were a function of the genetic distance between the house finch *M. gallisepticum* isolate used for inoculation and the poultry isolate used for coating the microwells, then we should see a decrease in antibody levels with years since the jump from poultry into house finches, as *M. gallisepticum* diverged away from its poultry ancestor and adapted to its novel host. However, this was not the case and, in fact, antibody levels were instead found to increase as the isolate used for inoculation was sampled progressively later in the epidemic (see “Results” section).

That *M. gallisepticum*-specific antibodies are associated not only with the level of resistance of the host population, but also with the level of pathogen virulence, sheds light on previous findings in this system. By inoculating house finches from resistant populations with four *M. gallisepticum* isolates, Grodio and colleagues^29^ found that the highest antigen-specific serum and lachrymal antibody levels were produced in response to the isolate that caused the most severe signs of conjunctivitis, and lowest following exposure to the isolate that caused the least. Our results confirm that variation in pathogen virulence can shape levels of *M. gallisepticum*-specific antibodies, although the mechanism by which it does so remains to be determined.

A greater antibody response has been found to be associated with increased resistance to *M. gallisepticum* infection in poultry. For example, vaccination of chickens with an avirulent strain of *M. gallisepticum* (GT5) conferred secondary immunity to a subsequent infection with the virulent strain R_low_, with vaccinated chickens displaying lower tracheal lesion scores than sham-immunised controls, as well as a thickening of the trachea due to the infiltration of leukocyte cells^30^. This reduced lesion and inflammatory response of GT5-vaccinated chicken was associated with a *M. gallisepticum*-specific serum IgY response that was both significantly more rapid (as early as 4 days post-challenge) and reached higher levels than that of sham-immunised controls. In our study, none of the finches used had previously been exposed to the pathogen, which means that they were all mounting primary, rather than secondary, immune responses to infection. Nevertheless, house finches from populations that have evolved resistance through immune clearance mounted similarly higher serum *M. gallisepticum*-specific IgY responses at 14, 28 and 35 days post-inoculation than finches from susceptible populations. This suggest that the antibody response contributes to shaping resistance upon first and secondary exposure to *M. gallisepticum*. However, our lack of  evidence for an association between antibody levels and subsequent declines in pathogen load also suggests that antibodies do not play a central role in the immune clearance process.

That antibodies play no or a limited role in pathogen clearance has previously been suggested for other mycoplasma species^31^. Antigen-specific antibody levels may not mediate clearance if antibodies serve other protective functions or if the pathogen is able to avoid antibody detection or binding. There are three lines of evidence regarding why such processes could occur during *M gallisepticum* infection. First, in poultry, antigen-specific antibodies may be more important in preventing the establishment of *M. gallisepticum* infection than in mediating its subsequent clearance. Antigen-specific IgYs present at the infection site (i.e. the respiratory mucosa) and either produced in situ or originating from serum, are indeed thought to mediate immunity to *M. gallisepticum* by preventing bacterial attachment to host tissues^32, 33^. Second, *M. gallisepticum* may be able to avoid antibody detection. Antibodies are thought to have limited protective benefits against intracellular pathogens, with infections by such pathogens thought to be better controlled through cell-mediated immune responses^34^. In accordance, *M. gallisepticum* has been shown capable of invading avian cells and persisting in the intracellular environment^35^, and the jump from poultry into house finches was associated with an increased ability to do both^36^. Third, *M. gallisepticum* may be able to prevent antibody binding. *Mycoplasma* spp. have been shown to possess a specific two-protein system (Mycoplasma Ig binding protein and Mycoplasma Ig protease; MIB-MIP) that captures and cleaves IgYs^37^. Homologs of the MIB-MIP system were found in *M. gallisepticum*^37^, but whether this system allows the pathogen to evade detection by house finch antibodies remains to be tested. Further work is required to understand the precise function of antigen-specific serum antibodies in mediating the resistance that house finches have evolved to *M. gallisepticum*.

In conclusion, we show that antibody production in response to a bacterial pathogen increases positively with levels of both host resistance and pathogen virulence, but fails to be associated with subsequent declines in pathogen load. Our results stress the need for caution when using a limited number of measures of immune activity as estimates of host resistance, even when such measures correlate with variation in disease severity. Further studies are needed to test the degree to which antibody levels confer protection against infection or reflect infection severity in natural populations of hosts, as well as to determine the extent to which our results can be generalised to other pathogens, particularly non-mycoplasma ones.

## Methods

### Capture, housing and experimental inoculation

In summer 2015, wild hatch-year house finches were trapped from urban and suburban sites in an area of Arizona where finches are known to have never been exposed to *M. gallisepticum* (i.e. susceptible populations)^38^. Birds were weighed, banded, and immediately brought back to Arizona State University, where they were housed in an indoor aviary for the remainder of the experiment^21^. During the same time period, we also caught hatch-year house finches from urban and suburban sites in Alabama. House finches in Alabama have been exposed to *M. gallisepticum* since initial disease outbreak and have evolved resistance. Birds were immediately brought back to aviaries at Auburn University^21^. In all birds from Arizona and Alabama, the lack of past and current infection with *M. gallisepticum* was verified by testing for the presence of: (1) *M. gallisepticum*-specific antibodies using the serum plate agglutination assay^16^, and (2) *M. gallisepticum* bacteria using PCR amplification of *M. gallisepticum* DNA in choanal swabs^39^. As expected, none of the birds tested positive for either test in Arizona^22^. Birds positive for either test in Alabama were released immediately; this ensured that: (1) none of the birds had any *M. gallisepticum-*specific IgYs in circulation before experimental onset; (2) antibody responses measured in this study were primary (not secondary) responses to infection; and (3) individuals remaining were not a biased sample of the population (i.e. those that were resistant to the infection). All birds from Arizona (N = 59; 29 females, 30 males) and all birds from Alabama that tested negative for both tests (N = 53; 29 females, 24 males) then underwent a > 30-day quarantine period. During this time, and although none of the birds displayed any sign of infection with other diseases, they were all treated prophylactically for *Trichomonas gallinae* with carnidazole (Spartrix, Janssen/Elanco) and *Isospora* spp. with sulfadimethoxine. At the end of this quarantine period, all birds from Alabama were transported in an air-conditioned vehicle to the aviary at Arizona State University. Care was used to minimise travel time (< 30 h), movement and stress to the birds; food and water was provided ad libitum throughout the trip and the birds were regularly checked for any signs of distress or injury. The results obtained in this study are unlikely to be impacted by automobile travel, as the population-difference in the response to infection measured in this experiment was equivalent to the one detected in an earlier study conducted at Auburn University (Alabama), and for which finches caught in Arizona had been driven the same distance in the opposite direction to Alabama^20^. Following the arrival of birds from Alabama at Arizona State University, all birds were allowed to acclimate for one additional month prior to experimental onset and provided with ad libitum food and water throughout. All birds were weighed (± 0.01 g) at the start and end of the experiment using a top-pan balance.

Each house finch was inoculated with one of 55 M*. gallisepticum* isolates via eye drops^21^. Isolates were originally obtained from naturally infected, wild-caught house finches and collected over a 20-year period across 8 different states in the eastern U.S. (mainly from Alabama)^21^. None of the isolates had been passaged in culture more than 3 times^40^ and were stored at − 80 °C. Isolates were administered via 20 μL of culture containing 1 × 10^4^ to 1 × 10^6^ colour changing units/mL of *M. gallisepticum* in both eyes, which corresponds to concentrations ranging from 4.1 × 10^5^ to 3.0 × 10^6^ bacterial cells/μL (average ± SE = 1.4 × 10^6^ ± 0.6 × 10^6^ bacterial cells/μL)^23^. We verified that variation in the number of bacterial cells inoculated (i.e. inculation dose) did not affect antibody levels on any of the three days they were measured (linear models; antibodies at 14 days post-inoculation (dpi): estimate ± SE = − 2.0 × 10^–9^ ± 1.6 × 10^–9^, t_1,93_ = − 1.4, *p* = 0.18; antibodies at 28 dpi: estimate ± SE = − 2.7 × 10^–9^ ± 2.8 × 10^–9^, t_1,94_ = 1.0, *p* = 0.34; antibodies at 35 dpi: estimate ± SE = 3.7 × 10^–10^ ± 2.0 × 10^–9^, t_1,94_ = 0.2, *p* = 0.86). The experiment was stopped at 35 dpi and all birds were euthanized as stipulated by home office licensing. Protocols were approved by Institutional Animal Care and Use Committees (IACUC) at Auburn University (permit # PRN 2015-2721) and Arizona State University (permit #15-1438R), by Institutional Biological Use Authorizations to Auburn University (# BUA 500), and by the University of Exeter’s ethics committee. The experiment was performed in strict accordance with relevant guidelines and regulations, including the USA federal regulations of the Animal Welfare Act (AWA, 7 USC 2131), the Institutional Animal Care & Use Committees, and the Animals (Scientific Procedures) Act 1986 (UK). This study is reported in accordance with ARRIVE guidelines (https://arriveguidelines.org).

### Pathogen load

Pathogen load was measured by quantitative amplification of *M. gallisepticum* DNA from conjunctival and tracheal swabs obtained at 8, 14, 21 and 28 dpi^23^. DNA was extracted using a QIAGEN DNeasy Blood and Tissue Kit according to manufacturer’s protocols. Multiplex quantitative PCRs for *mgc2* and *rag1* in each sample was then conducted using an Applied Biosystems StepOnePlus Real-Time PCR system^23^. Reactions were run in 20 µL volume containing: 2 µL of sample genomic DNA template, 1 µL each of 10 µM mgc110-F/R and rag1-102-F/R primers (total 4ul), 0.5 µL each of 10 µM Mgc110-JOE and Rag1-102-6FAM fluorescent hydrolysis probes (total 1 µL), 10 µL of 2× qPCRBIO Probe Mix HI-ROX (PCR BIOSYSTEMS) and 5 µL Nuclease-free water (Ambion). Reactions were then run at 95 °C for 3 min, followed by 45 cycles of 95 °C for 1 s and 60 °C for 20 s. Samples were run in duplicate alongside serial dilutions of plasmid-based standards. Amplification data was exported to LinRegPCR v.2017.1 for calculation of individual reaction efficiencies and quantification of low-amplification samples^41, 42^; between-run variation was normalised using Factor qPCR v.2016.0^43^, with plasmid standard serial dilutions used for factor correction. Pathogen load was then determined as the number of *M. gallisepticum* cells divided by the number of house finch cells to control for variation in sampling efficiency^44^.

### Antibody levels

At 14, 28 and 35 dpi, ~ 75 μL of whole blood was taken by brachial venipuncture and serum separated through centrifugation at 2000XG for 15 min at 4 °C before being stored at -80 °C. Goat Polyclonal Passerine IgY-heavy and light chain Antibody (Bethyl Laboratories Inc., TX, USA) was diluted to 1:1000 dilution according to manufacturer protocol. The prepared antibody was then conjugated to a visible proprietary HRP (horse radish peroxidase) ligand for visualization in enzyme-linked immunosorbent assay (ELISA), according to instructions accompanying the Lighting-Link HRP Conjugation Kit (Innova Biosciences Ltd., Cambridge, UK) for an antibody:HRP molar ratio of 1:4. *M. gallisepticum*-specific antibody detection was then performed with ELISA, with methods adapted from^10, 28^. Finch serum samples were diluted to 1:2400 in 1× sample conjugate diluent (Affinitech LTD, USA) and 100 μL of each sample was added to plate microwells coated with inactivated whole *M. gallisepticum* of poultry origin (Affinitech LTD). A standard curve of pooled sample was run on each plate in a twofold dilution series from 1:200 to 1:6400. Samples were run in duplicate, and care was taken to ensure that duplicates were distant from one-another on the plate to minimise variation caused by evaporation and edge effects. Plates were washed three times in 1× wash buffer (Bethyl Laboratories Inc.), before adding 100 μL of the prepared HRP-conjugated Passerine IgY-heavy and light chain antibody, diluted to a 1:10,000 concentration with sample conjugant diluent (Bethyl Laboratories Inc). After incubation for one hour in darkness, the plate was washed three times and 100 μL of TMB one component HRP microwell substrate (Bethyl Laboratories Inc.) was added to each well and incubated for 15 min in darkness. The reaction was stopped with 100 μL of 0.18 M H_2_SO_4_ and the plate was read on a plate reader at 450 nm within 30 min of stopping the reaction.

### Statistical analyses

All statistical analyses were conducted in R 3.3.2^45^. We used the package lme4^46^ to perform linear mixed models. The significance of explanatory terms were assessed by comparing models with and without each term using likelihood ratio tests. Figures were produced using the package ggplot2^47^. Out of the 112 birds experimentally-inoculated, we removed 4 that died during the course of the experiment due to incomplete data. To create a fully balanced paired design, we then considered only the isolates that were each inoculated into one bird from resistant populations and one from susceptible populations, and hence removed a further 8 birds from the analyses. We thus analysed data from a total of 50 pairs of birds (resistant populations: 26 females and 24 males; susceptible populations: 25 females and 25 males), each inoculated with one of 47 pathogen isolates (note that 3 isolates—2 from 1995 and one from 2007—were inoculated into 2 pairs of birds each).

Antibody levels on all days combined were natural log transformed, and antibody levels at 28 and 35 dpi were square root transformed, to fit a normal distribution. To determine whether antibody levels varied over the course of the infection, we ran a linear mixed model with ln(antibody level) as the response term, day post-inoculation (dpi) as the explanatory term, and pathogen isolate identity as the random term. Tests of the repeatability of antibody levels within individuals were conducted using the rpt function^48^, with ln(antibody levels) as the response term and house finch identity as the explanatory term. We tested the associations between antibody levels and sex or body mass using 3 linear models with antibody levels at 14 dpi or sqrt(antibody levels at 28 or 35 dpi) as the response term and with sex and body mass at the start of the experiment as explanatory terms (note: inclusion of isolate identity as a random term did not qualitatively affect the outcome of those models).

To test whether pathogen load predicted antibody levels, we ran linear mixed models with either antibody levels at 14 dpi or sqrt(antibody levels at 28 or 35 dpi) as the response term, and with pathogen load at 8, 21 or 28 dpi as an explanatory term; sex was included as a cofactor and pathogen isolate identity as a random term to maintain the paired design in the model. To then test the contribution of variation in host resistance and/or pathogen load in driving any association between bacterial load and antibody levels, we re-ran those models including host resistance status (resistant vs. susceptible populations) and the year of pathogen sampling (1994–2015) as additional explanatory terms. We used host resistance status to test for an effect of host resistance, and year of pathogen sampling for an effect of pathogen virulence, rather than metrics taken during the infection (e.g. clinical symptoms severity, pathogen load), because such metrics are unavoidably shaped by both host resistance and pathogen virulence. Finally, to determine whether antibody levels shaped changes in pathogen load, we ran linear mixed models with the difference in pathogen load between 21 and 14 dpi or between 28 and 14 dpi as the response term, and with antibody levels at 14 dpi, pathogen load at 14 dpi and their interaction as explanatory terms; sex was included as a cofactor and pathogen isolate identity as the random term.

## Supplementary Information

Supplementary Figure S1.

## Data Availability

Data reported in this paper have been deposited in Dryad Digital Repository 10.5061/dryad.x69p8czht.
