# Supplementary material for: Levels of pathogen virulence and host resistance both shape the antibody response to an emerging bacterial disease
Source: Sci Rep. 2021 Apr 15;11:8209. doi: 10.1038/s41598-021-87464-9 (PMC8050079; doi:10.1038/s41598-021-87464-9)

Gates et al.

Supplementary Material

Fig S1. Within and among-individual variation in antibody levels over the course of the infection. We measured antibody levels (in ELISA Units (EU)/ml) at 14, 28 and 35 days post-inoculation (dpi). We show the best fit regression lines for each individual; points represent raw data. Figure is provided for illustrative purposes only, as fitting robust regressions through 3 measurements points is not possible.

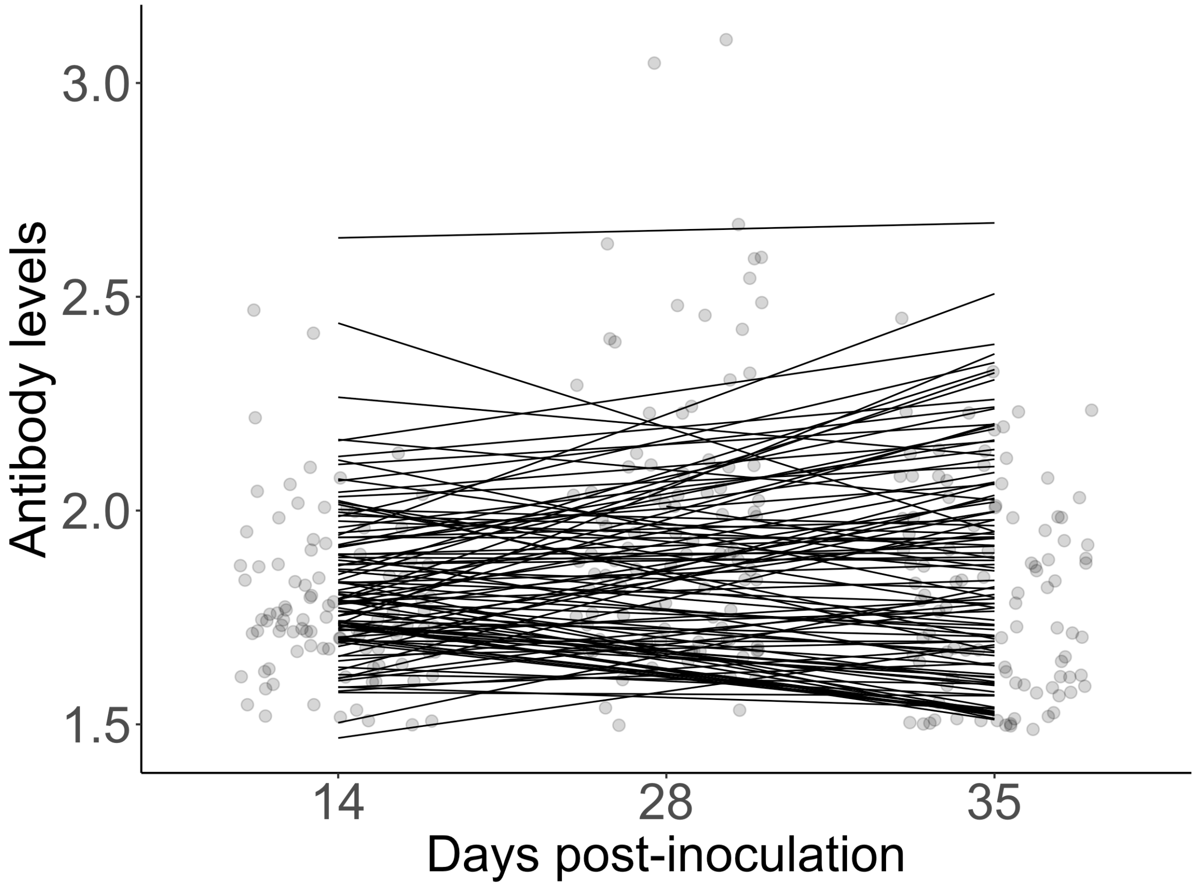

Supplement: Supplementary file 1 — Supplementary Figure S1. [file 41598_2021_87464_MOESM1_ESM.pdf]
